# Nurses’ mental health and COVID‐19 pandemic: Is there any approach?

**DOI:** 10.1002/nop2.930

**Published:** 2021-05-12

**Authors:** Khashayar Maroufi, Rashin Razavi

**Affiliations:** ^1^ Faculty of Physical Education and Sport Sciences University of Tehran Tehran Iran; ^2^ Faculty of Sport Sciences and Health Shahid Beheshti University Tehran Iran

The sudden and widespread outbreak of the novel coronavirus disease (COVID‐19) has caused many problems in all the sections of the societies (Liu et al., 2020). In the last few months, the increase in confirmed and suspected cases and deaths due to COVID‐19 in the world, and the imposition of more social restrictions in most countries has caused several psychological disorders among healthcare workers (HCWs) and the general population (Maroufi, 2021). Among HCWs, the frontline workers such as nurses and doctors who are involved directly in handling these patients are at greater risk than others (Spoorthy et al., 2020). With the rapid increase in the number of patients, doctors and nurses have to face enormous workload and high‐risk infections, which may lead to mental health problems such as anxiety, depression (Liu et al., 2020) or insomnia (Walton et al., 2020). This psychological distress caused by acute infectious diseases may persist for a long time and even lead to post‐traumatic stress disorder (PTSD) (Xiong et al., 2020).

Investigations demonstrated that several factors have such adverse psychological consequences among HCWs, including excessive work hours, depletion of personal protection equipment, over‐enthusiastic media news, feeling insufficiently supported, lack of specific drugs and the infection rate among medical staff (Lai et al., 2020; Spoorthy et al., 2020). There are many similarities in psychological symptoms between the COVID‐19 pandemic and the SARS or Ebola outbreak among HCWs (Lai et al., 2020). Moreover, it has been reported that nurses have higher anxiety and depressive symptoms than doctors (Spoorthy et al., 2020). On the other hand, some strategies, including the Internet, telephone or application‐based counselling or intervention, have been developed and administered by psychological health institutions to improve HCW’s mental health during the COVID‐19 pandemic (Lai et al., 2020). But the question is, along with counselling or interventions for nurses, doctors and medical staff, is there any approach that can be practical? We should first review the statistics of reports of psychological problems of health workers during the COVID‐19 pandemic.

Many studies have been conducted during this time. In a study, 1,257 HCWs completed questioners. Results showed that 50.4% of the HCWs suffered from depression, 44.6% from anxiety, 34.0% from insomnia and 71.5% from distress (Spoorthy et al., 2020). In another investigation, using Zung's self‐rating depression scale (SDS) and Zung's self‐rating anxiety scale (SAS), doctors and nurses showed experiencing clinically significant depressive symptoms (Liang et al., 2020). Kang et al. (2020) reported that among 994 medical and nursing staff in Wuhan, 36.9% had subthreshold mental health disturbances, 34.4% had mild disturbances, 22.4% had moderate disturbances and 6.2% had severe disturbances (Kang et al., 2020). Xiong et al., (2020) reported the outcome of a cross‐sectional survey among 223 nurses. Results showed the prevalence of anxiety and depression symptoms were 40.8% and 26.4%, respectively. In addition, they reported that self‐efficacy was negatively correlated with anxiety (*r* = −.161) (Xiong et al., 2020).

## 1 PHYSICAL ACTIVITY

Studies focused on the psychological effects of the COVID‐19 pandemic among HCWs. Some of which declared that health authorities should consider the conditions and methods to provide psychological support to both patients and HCWs. These methods may consist of using the Internet, telephone or web applications, to assess and monitor stress, depression and anxiety (Spoorthy et al., 2020). Increasing awareness, utilizing appropriate protective equipment or reducing work hours are suggested as practical approaches to improve HCW's mental health circumstances (Kang et al., 2020; Lai et al., 2020; Liang et al., 2020; Liu et al., 2020; Walton et al., 2020; Xiong et al., 2020).

We want to look at this issue from another perspective. Previous studies, American Sports Medicine Association (ACSM) and World Health Organization (WHO) have suggested regular physical activity to eliminate inappropriate mental conditions. Regular exercise and physical activity can alter the prevention and treatment of anxiety and depression (Maroufi, 2021). Regular physical activity regulates brain‐derived neurotrophic factor (BDNF), which increases cognitive function and the ability to face depression and anxiety (Hu et al., 2020). Studies showed that all types of exercise, such as aerobic, resistance or high‐intensity training, effectively reduced psychological disorders (Maroufi, 2021). Randomized controlled studies have illustrated that exercise is associated with an anti‐depressant effect (Martinsen, 2008). A meta‐analysis reported that using the Hamilton rating scale for depression, regular exercise and physical activity individuals diagnosed with clinical depression or those who do not respond to medication can be treated (Nagata et al., 2020).

The intensity, volume and frequency are the other remarkable variables to set up an exercise programme based on ACSM and WHO guidelines (Table 1). Moreover, the COVID‐19 pandemic‐related psychological distress is expected to have an improper impact on nurses, doctors and medical staff's physiological health. Anxiety and depression, despite psychological effects, may cause a sense of passivity and withdrawal, which has unpleasant consequences related to physiological markers during the pandemic (Maroufi, 2021). The primary adverse effects of lack of appropriate physical activity (Maroufi, 2021) and workplace environmental stressors (Shechter et al., 2020) are obesity, cardiometabolic syndrome and systemic inflammation, contributing to further health risks during the COVID‐19 pandemic. Due to the COVID‐19 pandemic, medical staff and HCWs have faced the magnitude of death of patients than usual; hence, the risk of post‐traumatic stress disorder (PTSD) has already arisen (Walton et al., 2020). Studies illustrated exercise interventions, regardless of the type or intensity, may reduce PTSD significantly (Hegberg et al., 2019). However, there is no direct evidence that shows the physical activity can alter the psychological circumstances in nurses, doctors or medical staff during the COVID‐19 pandemic; it seems, based on pre‐pandemic studies, following regular exercises and physical activities may improve their situations.

**TABLE 1:** Recommended effective physical activity for psychological indices’ changes and weight loss

Weight loss		Physical activity
No significant weight loss		>150 min. week^−1^
2–3 kg.week^−1^		150–225 min. week^−1^
5–7.5 kg.week^−1^	225–420 min. week^−1^	
Weight stabilization after a period of weight loss	200–300 min. week^−1^	

## 2 CONCLUSION

In conclusion, along with the psychological care of nurses, doctors and medical staff, setting up regular physical activity may have positive outcomes for the psychological health of HCWs during the COVID‐19 pandemic. Although the vaccination has been started globally, keeping the mental health of HCWs in mind, we can continue to fight against COVID‐19 stronger than past.

## CONFLICT OF INTEREST

There is no conflict of interest.

## AUTHOR CONTRIBUTION

Equal contributorship.
